# Novel humanized monoclonal antibodies for targeting hypoxic human tumors via two distinct extracellular domains of carbonic anhydrase IX

**DOI:** 10.1186/s40170-022-00279-8

**Published:** 2022-02-02

**Authors:** Miriam Zatovicova, Ivana Kajanova, Monika Barathova, Martina Takacova, Martina Labudova, Lucia Csaderova, Lenka Jelenska, Eliska Svastova, Silvia Pastorekova, Adrian L. Harris, Jaromir Pastorek

**Affiliations:** 1 MABPRO, a.s., Dubravska cesta 2, 841 04 Bratislava, Slovakia; 2 Department of Tumor Biology, Institute of Virology, Biomedical Research Center, Slovak Academy of Sciences, Dubravska cesta 9, 84505 Bratislava, Slovakia; 3 Department of Oncology, University of Oxford, Old Road Campus Research Building, Roosevelt Drive, Oxford, OX3 7DQ UK

**Keywords:** Carbonic anhydrase IX, Hypoxia, Humanized antibody, Tumor microenvironment

## Abstract

**Background:**

Hypoxia in the tumor microenvironment (TME) is often the main factor in the cancer progression. Moreover, low levels of oxygen in tumor tissue may signal that the first- or second-line therapy will not be successful. This knowledge triggers the inevitable search for different kinds of treatment that will successfully cure aggressive tumors. Due to its exclusive expression on cancer cells, carbonic anhydrase IX belongs to the group of the most precise targets in hypoxic tumors. CA IX possesses several exceptional qualities that predetermine its crucial role in targeted therapy. Its expression on the cell membrane makes it an easily accessible target, while its absence in healthy corresponding tissues makes the treatment practically harmless. The presence of CA IX in solid tumors causes an acidic environment that may lead to the failure of standard therapy.

**Methods:**

Parental mouse hybridomas (IV/18 and VII/20) were humanized to antibodies which were subsequently named CA9hu-1 and CA9hu-2. From each hybridoma, we obtained 25 clones. Each clone was tested for antibody-dependent cellular cytotoxicity (ADCC) and complement-dependent cytotoxicity (CDC) activity, affinity, extracellular pH measurement, multicellular aggregation analysis, and real-time monitoring of invasion with the xCELLigence system.

**Results:**

Based on the results from in vivo experiments, we have selected mouse monoclonal antibodies VII/20 and IV/18. The first one is directed at the conformational epitope of the catalytic domain, internalizes after binding to the antigen, and halts tumor growth while blocking extracellular acidification. The second targets the sequential epitope of the proteo-glycan domain, does not internalize, and is able to block the attachment of cancer cells to the matrix preventing metastasis formation. In vitro experiments prove that humanized versions of the parental murine antibodies, CA9hu-1 and CA9hu-2, have preserved these characteristics. They can reverse the failure of standard therapy as a result of an acidic environment by modulating the TME, and both are able to induce an immune response and have high affinity, as well as ADCC and CDC activity.

**Conclusion:**

CA9hu-1 and CA9hu-2 are the very first humanized antibodies against CA IX that are likely to become suitable therapies for hypoxic tumors. These antibodies can be applied in the treatment therapy of primary tumors and suppression of metastases formation.

**Supplementary Information:**

The online version contains supplementary material available at 10.1186/s40170-022-00279-8.

## Introduction

Hypoxia is an intrinsic property of solid tumors defined as a condition where partial O_2_ pressure is below 10 mmHg [1, 2]. Hypoxic tumor microenvironment (TME), caused by angiogenic dysregulation and consequent disruption of the vascular network, leads to metabolic and genomic changes [3]. At the molecular level, adaptation of tumor cells to the hypoxic TME is largely mediated by the hypoxia-inducible factor (HIF) family of transcription factors [4]. HIF targets include genes encoding mediators of angiogenesis such as vascular endothelial growth factor (VEGF) and VEGF receptors, enzymes of the glycolytic pathway such as hexokinase 2, lactate dehydrogenase, and glucose transporters (GLUT-1, GLUT-3), as well as pH regulators including carbonic anhydrase IX (CA IX) [5–7].

CA IX is one of the best responders to low oxygenation because of its transcriptional regulation, driven mainly by HIF-1 that binds to a hypoxia-response element (HRE) consensus sequence localized near the transcription initiation site of the *CA9* gene [8]. CA IX is a highly active member of the family of carbonic anhydrases which differs from the other CA isoforms by a strong association with cancer, hypoxia-related expression pattern, acidic pKa optimum, and a unique proteoglycan-like domain (PG) protruding from the globular catalytic domain of the enzyme [9–13]. CA IX is functionally involved in diverse aspects of cancer development, including protection of cancer cell survival in conditions of hypoxia and acidosis, facilitation of cancer cell migration and invasion, and contribution to metastatic dissemination, homing, and growth of metastatic lesions [14–19].

Hypoxia is a hallmark of solid tumors that has been linked to increased tumor metastasis and poor prognosis in cancer patients [1, 2]. Hypoxic tumor microenvironment does not only enhance proliferation and invasiveness of tumor cells, but also allows them to evade the immune system and impair drug delivery, further increasing immunotherapy and chemotherapy resistance, conferring them a survival advantage [20]. The presence of hypoxic areas is closely correlated with tumor progression and propagation of more aggressive and stress-resistant subpopulations where CA IX plays a critical adaptive role. Therefore, targeting hypoxia-induced molecules such as CA IX has a high potential for therapeutic benefits [21–23]. Numerous published studies on the role of CA IX in tumor biology and its clinical value support the view that it can serve as a biomarker and/or a therapy target in diverse tumor types and settings [24, 25]. These studies provide arguments in favor of using CA IX for cancer immunotherapy. First, CA IX is associated with hypoxia, acidosis, and aggressive tumor phenotype and thus expressed in a situation when available immunotherapy often fails. Second, CA IX is a very stable protein localized on the cell surface, and therefore, it is accessible to antibodies binding to its extracellular domains.

In the past, a collection of eleven anti-CA IX murine monoclonal antibodies was generated and characterized by Zatovicova [26]. However, the clinical use of murine monoclonal antibodies in cancer patients is highly limited due to human anti-mouse antibody response (HAMA). Thus, we selected the two most promising antibodies for humanization in order to explore their full therapeutic potential.

Here, we describe the construction and characterization of anti-CA IX antibodies named CA9hu-1 and CA9hu-2, which are the humanized versions of the murine monoclonal antibodies VII/20 and IV/18, specifically binding to distinct extracellular domains of CA IX and exhibiting disparate capabilities to induce CA IX internalization. We show that the humanization process completely preserved the binding specificity and affinity of the original mouse antibody. In addition, CA9hu-1 and CA9hu-2 acquired desirable effector functions, especially the capability for strong ADCC, antibody-dependent cell-mediated phagocytosis (ADPC), and complement-dependent cytotoxicity (CDC) in in vitro assays with human cancer cells and human effector cells. We also demonstrate the ability of the new antibodies to block the function of CA IX in pH regulation and invasiveness of tumor cells. Clinical grade humanized antibodies CA9hu-1 and CA9hu-2 are now being produced for the first-in-human clinical trials.

## Materials and methods

### Construction and characterization of humanized antibody variants CA9hu-1 and CA9hu-2

Humanization of VII/20 and IV/18 murine antibodies was performed by Fusion antibodies (Belfast, N. Ireland). After the RNA isolation from the pellets of hybridoma cells, cDNA was created by reverse-transcription with an oligo(dT) primer. PCR reactions were set up using variable domain primers to amplify both the V_H_ and V_L_ regions of the monoclonal antibody DNA. The V_H_ and V_L_ cDNAs were cloned and analyzed by DNA sequencing. After the VII/20 and IV/18 murine antibodies variable domains were sequenced, the CDRs were identified using antibody-numbering systems from IMGT and Kabat [27, 28]. For optimal retention of CDR-loop conformation, both numbering systems were used to identify a combined IMGT/Kabat CDR sequence of the murine antibody. Sequence analysis of the murine antibody was followed by humanized variant alignment. CDRs of the murine V_H_ and V_L_ were grafted into the acceptor frameworks. The combination of five VH and five VL chains resulted in the generation of twenty-five humanized variants having humanized variable domains [marked in the following text as heavy (HC) and light (LC) chain] and human Ig constant domains.

### Cell culture and transfection

Human cells with endogenous, hypoxia-inducible expression of CA IX: BT-20 (ATCC HTB-19) derived from breast carcinoma, JIMT-1 human breast carcinoma cells (HMS LINCS Database ID: 51118), MDA-MB-231 breast carcinoma cells (ATCC HTB-26), MBA-MB-468 (ATCC HTB-132), HT1080-iRFP670 cells (human fibrosarcoma cell line), and 42MGBA human glioblastoma cancer cells (Cellosaurus CVCL_1798) were cultivated under standard conditions in Dulbecco’s modified Eagle’s medium (BioSera, Nuaille, France) supplemented with 10% fetal calf serum (BioWhittaker, Basel, Switzerland) and gentamicine (Sandoz, Holzkirchen, Germany) in humidified air containing 21% O_2_ and 5% CO_2_ at 37 °C and in hypoxic conditions at an anaerobic workstation (Ruskinn Technologies, Bridgend, UK) in a humidified atmosphere containing 1% O_2_, 5% CO_2_, 10% H_2_, and 84% N_2_ at 37 °C. Experiments described in this paper were performed with C33-a human cervical carcinoma cells (ATCC HTB-31) and B16 F0 (ATCC CRL-6322) mouse melanoma cells transfected with the full-length human *CA9* cDNA (C33-a CA IX; B16-CA IX) in pcDNA3.1+ plasmid [29]. Related mock-transfected cells served as negative controls. Transfections were performed using TurboFectTM transfection reagent (Thermo Fisher Scientific, MA, USA). To obtain stable polyclonal cell lines, transfected cells were subjected to selection in G418 for 2 weeks and then separated on magnetic beads (Dynabeads M-450 Tosylactivated, Invitrogen, CA, USA) coupled to the CA IX-specific M75 monoclonal antibody according to the manufacturer’s instructions. Separated cell subpopulations were expanded and CA IX expression was analyzed by flow cytometry, western blotting, and immunofluorescence.

### ELISA screening of humanized antibodies

#### Preparation of antigens for ELISA

Protein extract from human cervical cancer cells C33-a CAIX permanently transfected with the full-length CA9 cDNA (C33-a CA IX) was used as a screening antigen. Lysate from mock-transfected cells was used as a negative antigen control (C33-a neo). Proteins were extracted from the cell monolayer with RIPA lysis buffer (0.1% deoxycholate, 1% Triton X-100 and protease inhibitor cocktail in PBS). Protein concentrations were determined by bicinchoninic acid assay (Thermo Fisher Scientific, Waltham, MA USA) according to the manufacturer’s instructions and diluted to a final concentration of 0.2 mg/ml in PBS.

#### ELISA procedure

Fifty microliters of either CA IX-positive or CA IX-negative protein extract was coated on the surface of high binding microplate wells (Greiner bio-one) overnight at 37 °C. After washing with PBS-T (0.05% Tween-20 in PBS pH 7.2), 50 μl of all humanized variants (diluted to concentration 5 μg/ml in 10% FCS in PBS-T) was added and incubated for 2 h at room temperature. Peroxidase-labeled swine anti-human IgG (diluted 1:5000 in 10% FCS in PBS-T; Sigma-Aldrich, St. Louis, MO USA) was used as a detector. Parental IV/18 and VII/20 antibodies (marked as “mouse Ab”) as well as chimeric HC0LC0 antibody (having the murine variable domains and the human Ig constant domains) were used as reference samples. The results are expressed by O.D. values of absorbance measured at 492 nm.

### Differential ELISA

For the CA IX domain differential ELISA, the wells were coated with the following antigens: RIPA extract of C33-a cells permanently transfected with the full-length CA9 cDNA (C33-a CA IX ), del PG CA9 cDNA (ΔPG), and del catalytic domain CA9 cDNA (ΔCA) in pcDNA3.1+ plasmid diluted to final concentration 0.2 mg/ml in PBS and were then assayed as above.

### SPR procedure setup

Surface plasmon resonance (SPR) analysis of antibody-antigen interactions was performed using Biacore technology (R&D grade) by Biaffin GmbH & Co KG, in Kassel, Germany. Each humanized antibody variant was captured on the sensor chip, and recombinant human CA IX protein (rh CA IX; 42 kDa; R&D systems) was added to the buffer flowing over the chip. Affinity measurements were performed using Biacore T200 instrument, and settings details of quantitative interaction analysis between antibodies and rh CA IX protein were as follows: flow rate, 30 μL/min for kinetic interaction analyses; analysis temperature, 25 °C; analysis buffer; 10 mM HEPES pH 7.4; 150 mM NaCl; 3 mM EDTA; 0.05% Tween 20; sensor chips CM5; setup, preparation of an α-human Fc capture surface, reversible capturing of antibodies, interaction analyses with antigen 1.56–400 nM rh CA IX (42 kDa): and complete removal of the antibody-antigen complex.

### Immunofluorescence assay

C33-a CA IX cells (300,000 cells per Petri dish) were plated on glass coverslips 24 h before the experiment and cultivated in different conditions: pH 7.2; pH 6.6, normoxia, and hypoxia. Cells grown on glass coverslips were incubated with antibody (5 μg/ml) for 1 h at 37 °C, gently washed with PBS and fixed in ice-cold methanol at − 20 °C for 5 min. Nonspecific binding was blocked by incubation with PBS containing 1% BSA for 30 min at 37 °C. Cells were then visualized by an antihuman Alexa Fluor® 488-conjugated antibody (Invitrogen, CA, USA) diluted 1:1000 in the blocking buffer for 1 h at 37 °C. The nuclei were stained with DAPI (Sigma-Aldrich, MO, USA). Finally, the coverslips were mounted onto slides in the Fluorescent Mounting Media (Sigma-Aldrich, MO, USA), and analyzed by the confocal laser scanning microscope Zeiss LSM 510 Meta.

### Immunofluorescence internalization assay

C33-a CA IX, JIMT-1, BT-20, MDA-MB-468, MDA-MB-231, and C33-a neo cells (300,000 cells per Petri dish) were plated on glass coverslips 24 h before the experiment. The live cells were incubated with the antibody (50 μg/ml) diluted in a culture medium at 4 °C for 30 min to recruit the mAb to CA IX on the cell surface. Subsequently, the cells were washed to remove any unbound antibody, transferred to 37 °C for 3 h to induce internalization, or fixed in ice-cold methanol at − 20 °C for 5 min. At the end of the 3 h treatment period, the cells were washed and fixed. After blocking, the primary antibody was visualized using anti-human Alexa Fluor® 488 secondary antibodies (Invitrogen, CA, USA, 1:1000 in 1% BSA). Finally, the cells were mounted onto slides and analyzed by the confocal laser scanning microscope Zeiss LSM 510 Meta.

### ADCC

ADCC reporter assay was performed according to the manufacturer’s instructions using C33-a CA IX, C33-a neo cells, and cancer cells expressing CA IX induced by hypoxia (breast cancer BT-20 and JIMT-1 and glioblastoma 42MGBA). Cells (12,500 cells/well) were plated onto sterile 96-well plates and incubated in a culture medium overnight at 37 °C. Humanized antibody variants (CA9hu-1 or CA9hu-2) were diluted to 1 μg/ml in PBS, and 75,000 effector cells (according to the recommended effector: target ratio 6:1) were used per well. After 6 h of incubation, detection of firefly luciferase was performed using Bio-GloTM Luciferase Assay Reagent (Promega). The mixture of samples with ADCC assay buffer and effector cells without adding the humanized antibody is marked as “no Ab.” The mixture of samples without antibody and effector cells is marked as “no Ab, no EC” and serves as “plate background.” The results are expressed as luminescence in relative luminescence units (RLU). For EC_50_ determinations, target cells were incubated with a series of concentrations of antibodies, followed by the addition of ADCC Bioassay Effector Cells. The E:T ratio was 6:1. After 6 h of induction at 37 °C, Bio-Glo™ Luciferase Assay Reagent was added, and luminescence was determined using a GloMax®-Multi+ Luminometer. Data were fitted to a 4PL curve using the GraphPad Prism® software.

### ADPC

To evaluate the ability of humanized antibodies to mediate phagocytosis, ADCP Reporter Bioassay System (Promega) was applied. ADCP reporter assay was performed according to the manufacturer’s instructions using C33-a CA IX as well as C33-a neo cells. One day before analysis, 12,500 cells per well were plated onto a sterile 96-well plate and incubated in culture medium overnight at 37 °C. Humanized antibody variants CA9hu-1(HC4LC4) and CA9hu-2(HC4LC5) were diluted to 2 μg/ml in PBS, and 75,000 effector cells (according to the recommended effector: target ratio of 6:1) were used per well. After 6 h of incubation, detection of firefly luciferase was performed using Bio-Glo™ Luciferase Assay Reagent (Promega).

### CDC

Twenty-four hours before the assay, C33-a CA IX as well as C33-a neo cells were plated onto a sterile 96-well plate in a concentration of 20 × 10^4^ cells per well in 50 μl of DMEM culture medium with 10% FCS and incubated overnight in CO_2_ incubator with 5% CO_2_ at 37 °C. On the day of the analysis, CDC assay was performed using rabbit complement serum (BAG Healthcare). First, the culture medium was removed from each of the wells, and 50 μl of the fresh culture medium with 10% FCS and 50 μl antibody diluted to 5 μg/ml was added per well. Samples without antibodies were used as “no antibody” control. The mixture was incubated at room temperature for 5 min, and 10 μl of rabbit complement serum was added to each well, mixed, and cultured at a standard condition for 24 h. After incubation, cell viability was analyzed by CellTiter-Blue® Cell Viability Assay, Promega) according to the manufacturer’s instructions. The fluorescence was recorded with a 530-nm/590-nm (excitation/emission) filter set using a Bio-Tek Synergy HT microplate reader.

### Measurement of extracellular pH

Before the assay, C33-a CA IX cells were seeded onto 24-well plate HydroDish® (105,000 cells/well) and allowed to attach for 3–5 h. Subsequently, the culture medium was replaced with a medium containing lowered bicarbonate and serum (1 ml/well) to mimic conditions characteristic for tumors. We used DMEM medium (Sigma-Aldrich) supplemented with 4.5 g/l glucose, 22 mM NaHCO_3_, 1% FCS, 4 mM glutamine, and 1 mM pyruvate with the presence or absence of humanized antibodies CA9hu-1(HC4LC4) or CA9hu-2(HC4LC5) and control irrelevant IgG (50 μg/ml). Cells were incubated in hypoxia (1% O_2_, 5% CO_2_, 10% H_2_ and 84% N_2_, Ruskinn Technology, at 37 °C) for the next 48 h, and pH of culture medium was analyzed by non-invasive online pH monitoring using pH measuring device SDR SensorDish® Reader (PreSens Precision Sensing GmbH).

### Multicellular aggregation analysis

The non-ionic acid poly(2-hydroxyethyl methacrylate) (poly-HEMA; Sigma-Aldrich), which inhibits matrix deposition and cell attachment, was dissolved in 99% ethanol at 10 mg/ml. Six-well tissue culture plates were coated with 0.5 ml of poly-HEMA solution, allowed to dry, washed with PBS, and stored at 4 °C. C33-a CA IX cells (400,000 cells/well) were added to poly-HEMA-coated wells and cultured in the presence or absence of humanized antibody variant (30 μg/ml) for 24 and 72 h. To evaluate the ability of C33-a CA IX cells to form multicellular aggregates, images from either treated or untreated cells were acquired, and the accumulated pixel density was measured using the ImageJ software. At the end of the longer treatment (72 h), C33-a CA IX cells were recovered, centrifuged, and subsequently analyzed via flow cytometry using propidium iodide to stain dead cells.

### Real-time monitoring of invasion with xCELLigence system

The xCELLigence cell index impedance measurements were performed using the CIM-Plate16 in which each well is composed of upper and lower chambers separated by an 8-μm microporous membrane placed in the RTCA DP station according to the instructions of the supplier (Roche, Basel, Switzerland). Cells were trypsinized, resuspended at the density of 400,000 cell/ml in a serum-free medium, added to the Matrigel coating top chamber of the CIM-Plate, and allowed to invade towards the bottom chamber containing medium with 10% FCS as a chemoattractant. Invasion was measured as the relative impedance change (cell index) across microelectronic sensors integrated into the bottom side of the membrane. Invasion was monitored every 15 min for 60 h in hypoxic conditions (1% O_2_). For quantification, the cell index at the indicated time points was averaged from six independent measurements.

### In vivo experiments

NMRI-Foxn1^nu^ nu/nu female mice and C57BL/6J female mice (Charles River Laboratories, Inc.) were housed in SPF facility and used in accordance with the Institutional Ethics Committee guidelines under the approved protocols. The project was approved by the national competence authority—State Veterinary and Food Administration of the Slovak Republic (No. Ro. 4245/13-221 and 292/16-221 g)—in compliance with the Directive 2010/63/EU and the Regulation 377/2012 on the protection of animals used for scientific purposes. Mice were housed in groups of 3 randomized animals in individually ventilated IVC (Tecniplast) cages with wooden fiber bedding, at 20 ± 2 °C temperature, using natural light/dark cycle, with SNIFF diet, ad libitum access to food and water, with environmental enrichment by paper houses. The mice were subjected to regular monitoring with a humane endpoint. The number of mice was kept at minimum required to achieve statistical significance; in vivo study was designed based on thorough preceding in vitro experiments. Primary tumors were generated by a subcutaneous injection of a suspension of B16-FL-CA IX melanoma cells (5 × 10^5^ cells in 100 μl PBS) into the right and left upper flanks of NMRI-Foxn1nu nu/nu male mice (*n* = 3). During the experiment, the antibodies were intravenously administered (100 μg in 100 μL PBS) on the 0, 5th, 8th, 12th, and 15th day. Mice were sacrificed on the 16th day after inoculation. For the lung colonization assay, hypoxia pre-incubated HT1080-iRFP670 cells were treated or non-treated with IV/18 to block the PG-domain of CA IX protein. Cells were injected into the tail vein (1.5 × 10^6^/mouse) of NMRI nude mice (NMRI-Foxn1^nu^ nu/nu female mice, Charles River Laboratories, Inc., 10 mice/group). During the experiment, the antibodies were intravenously administered (50 μg in 100 μL PBS) every 3 days. After 12 days, mice were sacrificed. PBS-perfused lungs were ex vivo evaluated for the fluorescent signal emitted by metastasizing cancer cells using an IVIS system (In vivo Imaging System, Caliper Life Sciences).

### Statistical analysis

Continuous variables were expressed as mean ± SEM and evaluated either by ANOVA or by the Student *t*-test (between two groups). A *P* < 0.05 was considered significant (*).

## Results

### Anti-tumor effects of parental murine monoclonal antibodies

Excellent properties and characteristics of previously described anti-CA IX mouse monoclonal antibodies VII/20 targeting catalytic CA-domain and IV/18 specific to PG-domain [26] predetermined them as the basis for the generation of humanized antibodies for anti-cancer therapeutic application. Biological properties of the VII/20 mAb and its capacity to reduce tumor growth were evaluated earlier in the mouse xenograft model of HT29 colorectal carcinoma [30].

**Fig. 1:**
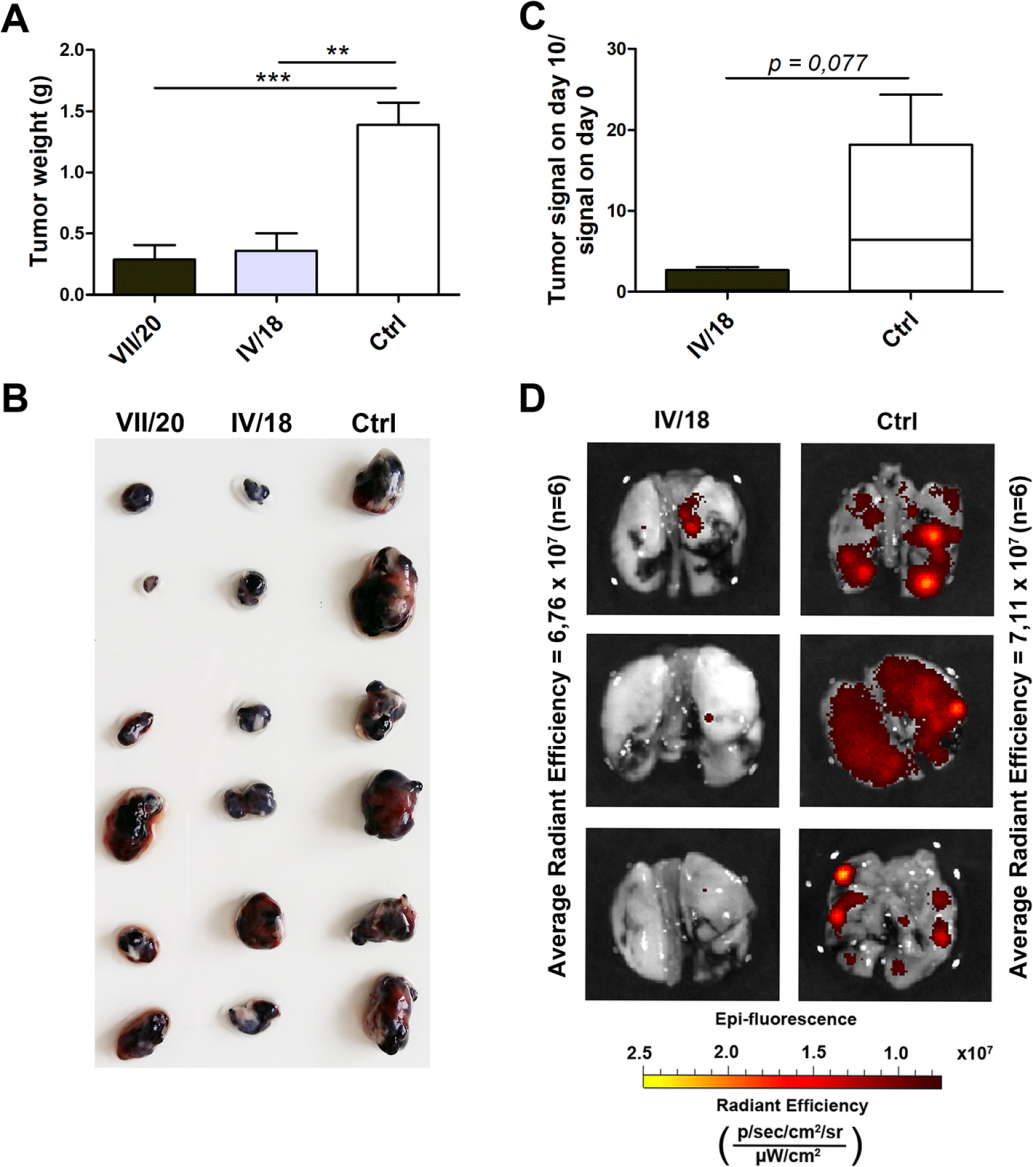
Anti-tumor effect of CA IX-specific mouse monoclonal antibodies in vivo. **A**, **B** Inhibition of tumor growth by antibodies IV/18 and VII/20 on B16-CA IX cell xenografts stably transfected with human CA IX in C57BL/6J mice model. **C**, **D** Inhibition of lung metastasis formation of hypoxia pre-incubated HT1080-RFP cells treated by CA IX-specific antibody IV/18 in tail vein colonization assay. Data in the graph represent the mean ± standard deviation values. Statistical significance of the differences between antibody-treated and non-treated animals was assessed using Student’s *t*-test (*P < 0.05, **P < 0.01, ***P < 0.001), *n* = 6

Here, we investigated the anti-cancer effect of both IV/18 and VII/20 antibodies on the growth of primary tumors in subcutaneous xenografts formed from mouse melanoma cells. 5 × 10^5^ mouse melanoma B16-F0 cells stably transfected with human CA IX and 50 μg of antibody were injected subcutaneously into the left and right flank region of mice. During the experiment, the antibodies were intravenously administered every 3 days. The results showed that both antibodies significantly reduced tumor weight when compared to the control, non-treated animal (marked as “Ctrl”) (Fig. 1A, B).

We further evaluated the ability of the antibodies to attenuate cancer cell extravasation and metastasis formation in a murine lung colonization model. Metastatic colonies of fluorescently tagged HT1080-RFP cells in murine lungs were imaged ex vivo 12 days after injection using the IVIS Caliper Imaging System, in which total radiant efficiency reflects the amount of cancer cells in murine lungs. Pre-incubation of HT1080-RFP cells with mAb IV/18 and subsequent administration of 3 doses of antibodies (50 μg/mouse) during 12 days after the initial tail vein injection (1.5 × 10^6^ cells per animal, 10 mice per group) caused a marked decrease in lung colonization by these cells (Fig. 1C). Since the metastatic colonies were evaluated ex vivo relatively shortly after the tail vein inoculation (12 days), reduced extravasation was apparently the main factor behind the decreased metastasis formation following treatment with IV/18 antibody (Fig. 1D).

### Humanization of VII/20 and IV/18 antibodies

Based on *in vivo* anti-tumor properties of CA IX-specific VII/20 and IV/18 murine monoclonal antibodies, we decided to generate their humanized versions for human anti-cancer therapy. Humanization of anti-CA IX antibodies was achieved by a complementarity-determining region (CDR) grafting approach. A number of human framework sequences (coming from mature human IgG from a human source) were identified and used as “acceptor” frameworks for CDR sequences. Thus, the humanized sequences are expected to be non-immunogenic and retain the canonical structure of the CDR-loops. DNAs coding for the amino acid sequence of humanized variants were synthesized and cloned into an appropriate mammalian expression plasmid. Twenty-five humanized variants of CA9hu-1 from the parental mouse antibody VII/20 and 25 variants of CA9hu-2 from the mouse antibody IV/18 were created comprising murine-derived CDRs and humanized heavy and light regions.

### CA IX-binding properties of the humanized CA9hu-1 and CA9hu-2 antibodies

An ideal antibody humanization should be capable of maintaining the specificity and affinity towards the antigen comparable with parental mouse immunoglobulin. However, the loss of specificity and affinity of an antibody to its specific target is the main problem of humanization [31]. For this reason, all antibody variants of CA9hu-1 and CA9hu-2 were screened for specific binding and affinity towards the human CA IX antigen by ELISA using antigens prepared from a stably transfected C33-a cell line expressing CA IX (C33-a CA IX) and parental mock-transfected C33-a cells without CA IX expression (C33-a neo). The results show that both humanized antibodies retained specific and effective binding similar to or exceeding that of the parental antibodies (Additional file 1: Fig. S1). In case of CA9hu-1 (Additional file 1: Fig. S1A), HC4 variants exhibited the highest binding efficiency against CA IX. Similarly, the highest binding efficiency was observed in case of HC3 and HC4 variants of CA9hu-2 antibody (Additional file 1: Fig. S1B).

To evaluate the CA IX domain-specificity of the humanized antibodies, the representative antibody variants CA9hu-1 (HC4LC4) and CA9hu-2(HC4LC5) were analyzed by ELISA against the CA IX protein with deletions in PG and catalytic domains, respectively. In line with the specificity of the parental antibodies, CA9hu-1 (HC4LC4) binds to the catalytic domain, while CA9hu-2 (HC4LC5) is directed against the PG domain (Additional file 1: Fig. S1C).

The antigen-binding affinity of all humanized variants was assessed by SPR using a Biacore instrument. The results of real-time monitoring of their binding kinetics with recombinant human CA IX protein are expressed as the equilibrium dissociation constant (*K*_*D*_). As shown in Additional file 2: Table S1, all antibody variants possess *K*_*D*_ values in a low nanomolar range (10^−7^–10^−9^) that is generally considered to be the range of high-affinity antibodies. Moreover, some antibody variants of CA9hu-1 (HC4LC1, HC4LC2, HC4LC3, HC4LC4, and HC4LC5) showed even higher affinity than the chimeric variant.

### Membrane CA IX binding and internalization of the humanized antibodies

**Fig. 2:**
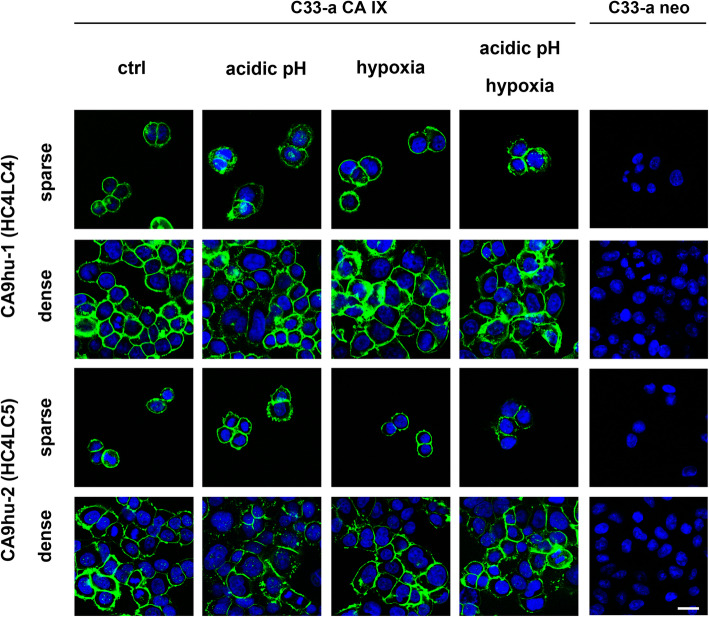
Immunofluorescence detection of CA IX in transfected C33-a CA IX cells. The cells were grown to confluence and incubated with representative monoclonal antibodies in different conditions then fixed by methanol followed by incubation with anti-human Alexa Fluor® 488-conjugated antibody. Both antibodies revealed plasma membrane CA IX-specific staining. Scale bar = 20 μm

There are several arguments in favor of CA IX being a suitable target molecule for cancer therapy. One of the strong reasons is the CA IX exposure on the cell surface and its accessibility to an antibody from the extracellular space. Indirect immunofluorescence demonstrated that both of the antibody variants CA9hu-1 (HC4LC4) and CA9hu-2 (HC4LC5) bound specifically to the CA IX antigen localized at the surface of transfected cells C33-a CA IX at 37 °C in sparse cell culture as well as in high cell density and also in acidic and hypoxic conditions (Fig. 2).

**Fig. 3:**
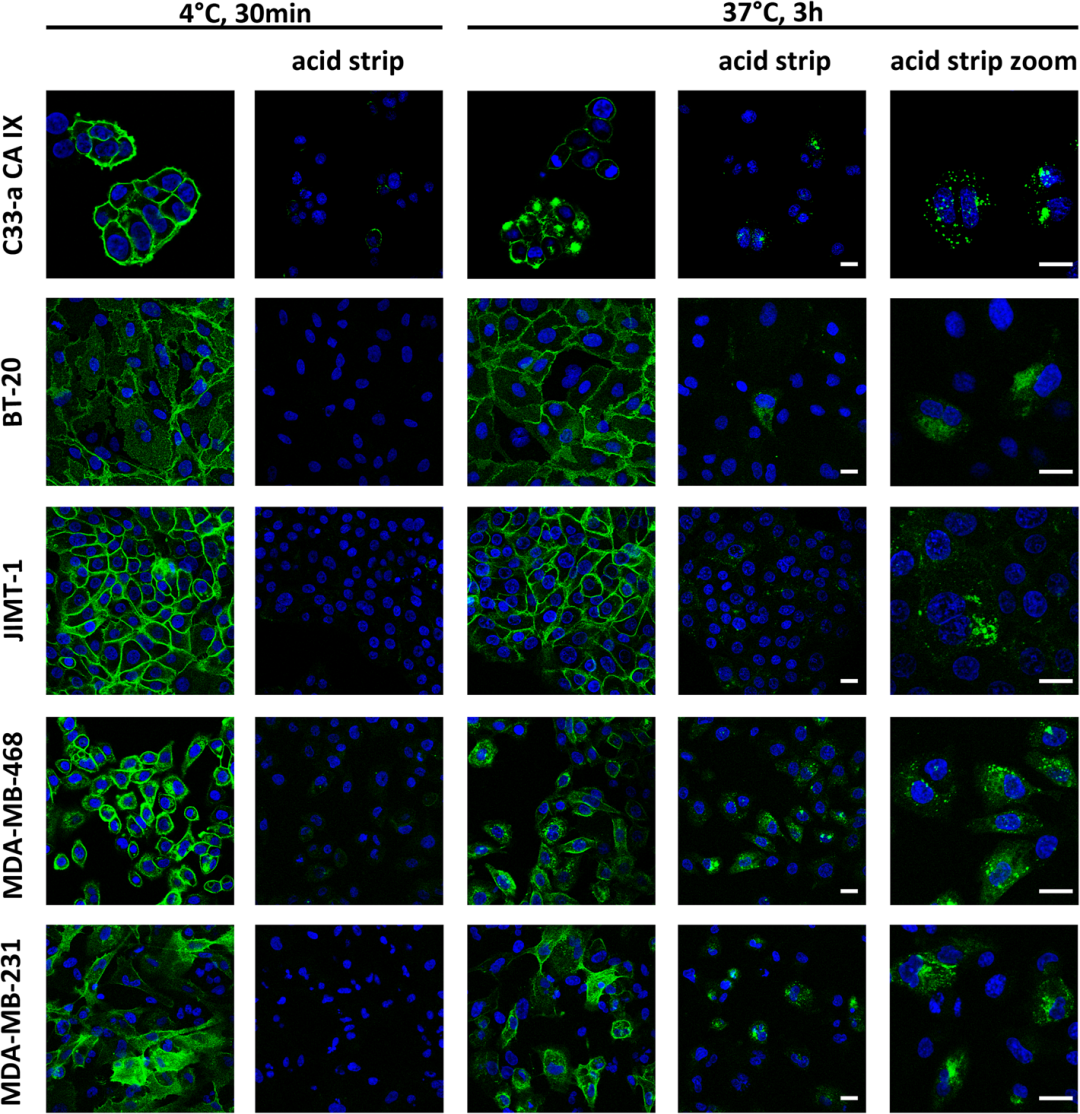
The analysis of humanized antibody variant CA9hu-1(HC4LC4) for its capacity to internalize into cancer cells. Visualization by confocal microscopy revealed punctuated intracellular staining signal after acid strip indicating internalization. Cells were incubated with MAb at 4 °C for 30 min and then in parallel at 4 °C (to prevent internalization) and 37 °C (to trigger internalization) for 3 h. After fixation with methanol, the cells were stained with anti-human Alexa Fluor® 488-conjugated antibody in order to visualize the MAbs. Acid strip before fixation was used to remove the membrane-bound antibody. Scale bar = 20 μm

Effective applications of monoclonal antibodies in cancer therapy rely on their ability to specifically target cancer tissues but, in some cases, also to enter the intracellular space via receptor-mediated internalization. Since the CA9hu-1 antibody was derived from the internalizing murine antibody VII/20 [30], we wanted to find out whether this capability was preserved during the humanization process. Using C33-a CA IX cells that express high levels of CA IX and employing also a collection of carcinoma cells with natural CA IX expression, we demonstrated that CA9hu-1 (HC4LC4) antibody directed to the catalytic domain of CA IX is able to induce the CA IX-mediated internalization (Fig. 3). The cells were allowed to bind mAbs at 4 °C for 30 min and incubated for 3 h at 37 °C to enable internalization. After washing off unbound antibodies, cells were fixed with methanol and treated with Alexa Fluor® 488-conjugated secondary antibody to visualize the primary mAbs. CA9hu-1 (HC4LC4) antibody was accumulated in the cytoplasm after incubation at 37 °C showing its internalization. We examined the internalization of CA9hu-2 under the same conditions. The fluorescence signal in the cytoplasm was extremely low (data not shown), it was almost undetectable.

### Cytotoxicity-inducing effector functions of humanized CA IX-specific antibodies

The main expected role of the new anti-CA IX humanized antibodies in cancer immunotherapy is to stimulate the host immune system to attack the CA IX expressing cancer cells. The underlying mechanisms include natural killer (NK) cell-mediated antibody-dependent cellular cytotoxicity (ADCC), macrophage-mediated antibody-dependent cell phagocytosis (ADCP), and/or complement-dependent cytotoxicity (CDC). We therefore examined the capacity of our humanized antibodies to induce these effector functions.

**Fig. 4:**
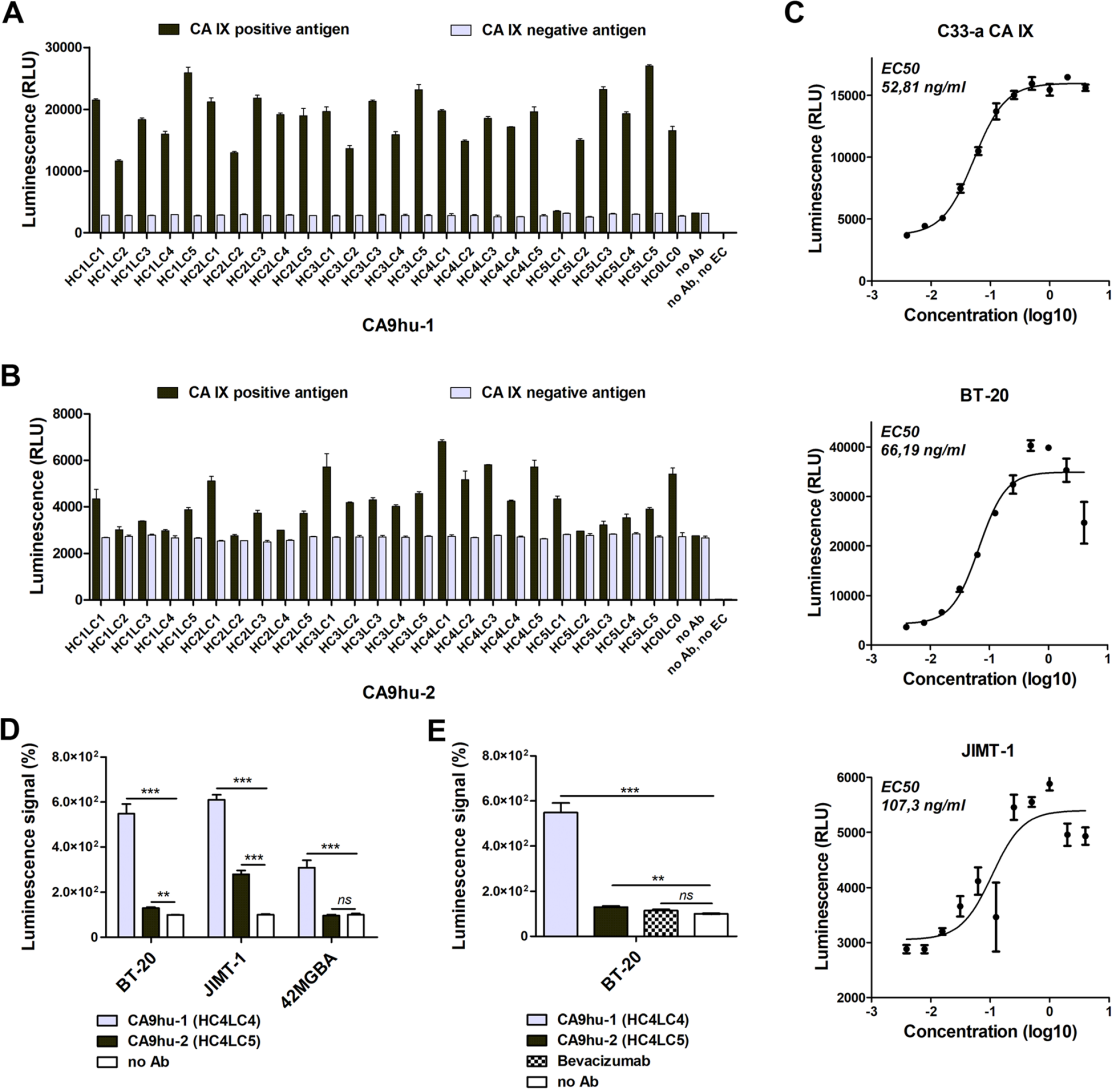
**A**, **B** Graphical representation of ADCC data. CA9hu-1 and CA9hu-2 antibody variant-dependent cell-mediated cytotoxicity. Comparison of CA IX-negative versus CA IX-positive target cell line samples, *n* = 2. **C** Fit curves determine EC_50_ of CA9hu-1(HC4LC4) antibody response using the GraphPad Prism® software. **D** Humanized antibody variants showed high ADCC activity in Reporter ADCC assay (Promega) tested on cells BT-20, JIMT-1, and 42MGBA naturally expressing CA IX in hypoxia. The signal of the control was set as 100%. **E** Humanized antibody variants showed high ADCC activity in comparison with irrelevant therapeutic antibody bevacizumab. This figure clearly shows that humanized antibody variants CA9hu-1 and CA9hu-2 retain the ability to activate the ADCC pathway and to mediate the cytotoxic effect on target cells expressing CA IX. Data in the graph represent the mean ± standard deviation values. Statistical significance of differences was assessed using Student’s *t*-test (*P < 0.05, **P < 0.01, ***P < 0.001), *n* = 3

The ability of humanized antibody variants to mediate the antibody-dependent cytotoxic effect was evaluated using ADCC reporter bioluminescence assay [32]. The assay employs engineered Jurkat cells stably expressing the FcγRIIIa receptor, V158 high-affinity variant, and NFAT (nuclear factor of activated T-cells) response element driving expression of firefly luciferase, as effector cells. The ADCC response was quantified through the luciferase production as a result of NFAT activation. As shown in Fig. 4A, B, CA9hu-1 and CA9hu-2 variants exhibited high luminescence signal and, thus, high cytotoxicity against C33-a CA IX expressing cells. As the RLU of samples were 100 times higher than the plate background RLU (marked as “no Ab, no EC cells”), there was no need to subtract plate background from the sample RLU. Although CA9hu-2 variants appeared to be less efficacious than CA9hu-1 antibodies, they still possess two times higher capacity to activate the cytotoxic pathway via ADCC against C33-a CA IX expressing cells than against C33-a neo cells.

To prove the antibody-dependent cell-mediated cytotoxicity on cancer cells naturally expressing CA IX, we analyzed TNBC cell lines BT-20 and JIMT-1 as well as glioblastoma cell line 42MGBA. In these cell lines, CAIX expression is induced by hypoxia. Figure 4C, D clearly shows that selected humanized antibody variants CA9hu-1 (HC4LC4) and CA9hu-2 (HC4LC5) in a lower extent can activate the ADCC pathway and mediate the cytotoxic effect on target cells expressing CA IX. The highest induction (> 25-fold) was observed in TNBC cells JIMT-1 and BT-20 after the treatment with CA9hu-1 (HC4LC4) antibody. Moreover, we compared the ADCC activity of novel humanized antibodies with a therapeutically used antibody bevacizumab, with a declared low ADCC activity. As can be observed in Fig. 4E, the CA9hu-1 antibody showed significantly higher ADCC activity compared to bevacizumab. In addition, we calculated the EC_50_ for both humanized variants. The EC_50_ value of CA9hu-1 variant response using C33-a CA IX, BT-20, or JIMT-1 target cells was 52 ng/ml, 66 ng/ml, and 107 ng/ml, respectively, which is comparable to EC_50_ of therapeutic antibodies trastuzumab and cetuximab (as declared by the ADCC assay manufacturer).

**Fig. 5:**
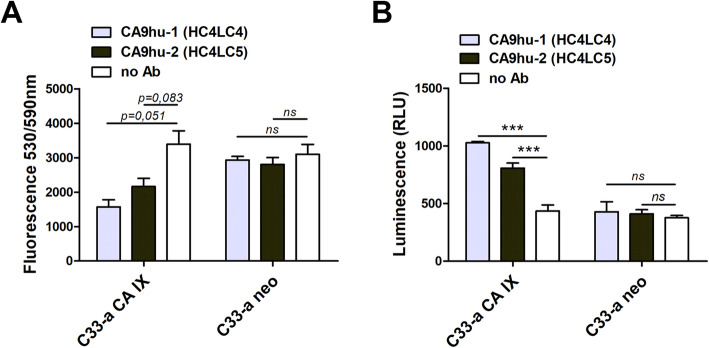
**A** The effect of selected humanized antibodies (CA9hu-1(HC4LC4) and CA9hu-2(HC4LC5)) on the viability of analyzed cells ± expressing CA IX (C33-a CA IX versus C33-a neo) in the presence of complement determined via Cell Titer Blue Viability Assay. Cancer cells incubated in the absence of humanized antibodies are marked as “no Ab.” Data in the graph represent mean ± standard deviation values. Statistical significance was assessed using Student’s *t*-test (*P < 0.05, **P < 0.01, ***P < 0.001), *n* = 2. **B** Graphical representation of ADCP data. CA9hu-1 and CA9hu-2 antibody variant-dependent cell-mediated phagocytosis. Comparison of CA IX-negative versus CA IX-positive target cell line samples. Statistical significance was assessed using Student’s *t*-test (*P < 0.05, **P < 0.01, ***P < 0.001), *n* = 3

The ability of humanized antibody variants to participate in complement-dependent cytotoxicity (CDC) was determined by incubation of rabbit serum with CA IX-positive C33-a CA IX cells, and C33-a neo cells in presence of tested antibody and recorded using cell viability assay after 24 h. Measured fluorescence data are shown in Fig. 5A. Complement binding and activation analysis showed that CA9hu-1 (HC4LC4) and also CA9hu-2 (HC4LC5) antibody variants can induce the cytostatic response of CA IX-positive C33-a CA IX cells in the presence of complement when compared to no-antibody control. This was not observed in CA IX-negative cell line C33-a neo. The results demonstrate that humanized variants of both CA9hu-1 and CA9hu-2 can be used to specifically distinguish and consequently mediate the cytotoxic effect on tumor cells expressing CA IX via CDC.

For in vitro analyses of the ability of humanized antibody variants CA9hu-1 (HC4LC4) and CA9hu-2 (HC4LC5) to mediate phagocytosis, we used an ADCP reporter bioluminescent cell-based assay, which measures the potency and stability of antibodies and other biologicals containing Fc domains that specifically bind and activate FcγRIIa. The assay uses engineered Jurkat T-cells expressing the FcγRIIa receptor, H131 high-affinity variant, and NFAT response element driving expression of firefly luciferase as effector cells. Thus, the ADCP mechanism of action is quantified through the luciferase production as a result of NFAT activation. The results are expressed as luminescence in relative luminescence units (RLU).

As shown in Fig. 5B, almost no phagocytic activity was observed when CA IX-negative C33-a neo cells were used as target cells independently of the presence or absence of CA IX-specific antibodies. The phagocytic potency was acquired after the incubation of CA IX-expressing cancer cells in the presence of humanized antibody variant CA9hu-1 (HC4LC4) (236%) and also CA9hu-2 (HC4LC5) (185%). These results demonstrate that both humanized antibody variants can be used to specifically recognize and consequently mediate phagocytosis of cancer cells expressing CA IX. Considering the fact that ADCP is an important mechanism of action of therapeutic antibodies, the phagocytic potency of the humanized antibody represents an extraordinary and beneficial property.

### Effects of CA9hu-1 and CA9hu-2 antibodies on biological functions of CA IX

Therapeutic approaches targeting CA IX have primarily focused on the development of specific monoclonal antibodies which are able to detect and kill tumor cells expressing CA IX in cooperation with the immune system. The second approach for targeting CA IX cancer cells involves the utilization of antibodies for blocking the functions of CA IX in tumor biology, especially its pro-survival role in protecting tumor cells in hostile acidic microenvironments and in adhesion–migration–invasion.

**Fig. 6:**
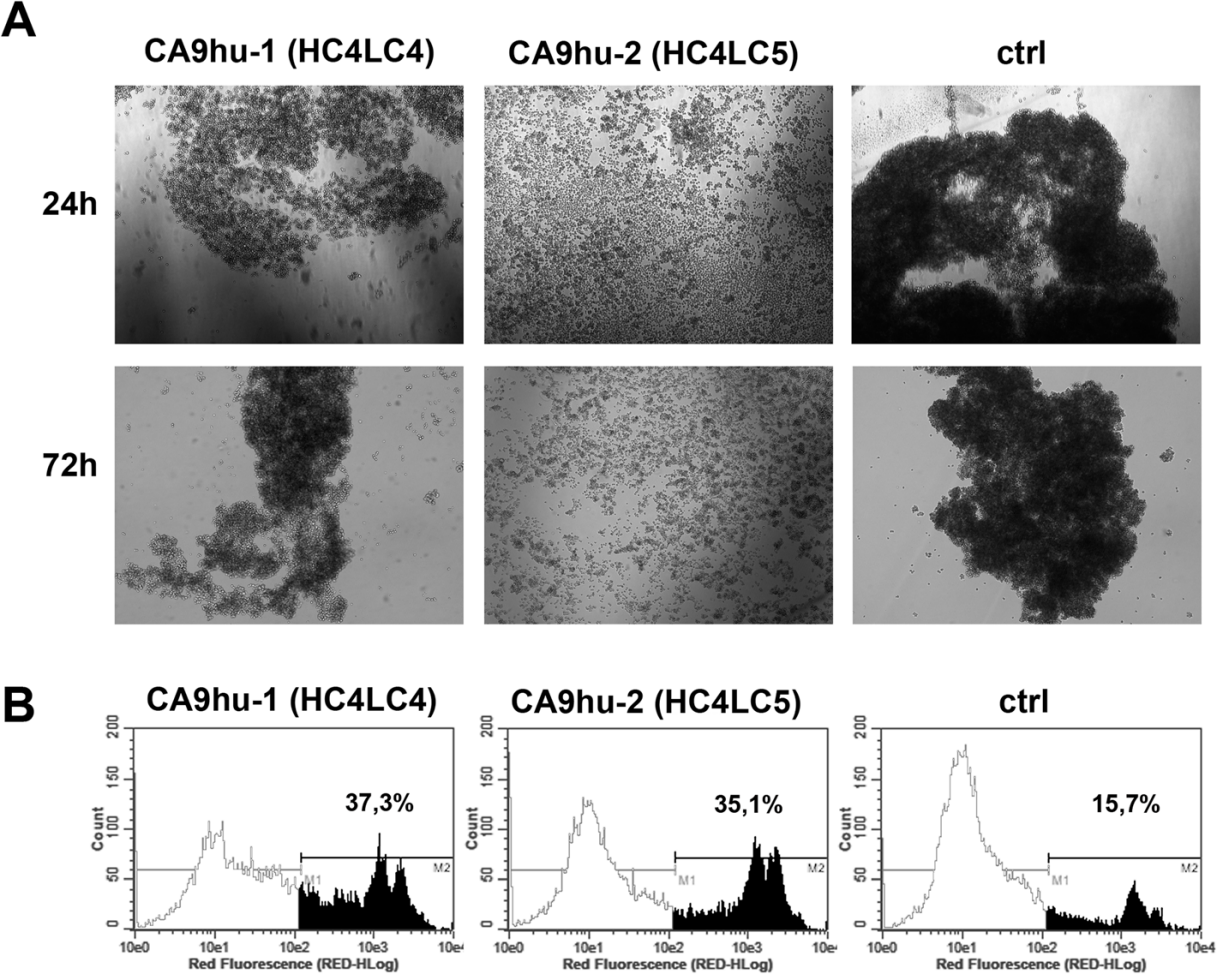
**A** Analysis of multicellular aggregation of C33-a CA IX with humanized antibody CA9hu-1 (HC4LC4) and CA9hu-2 (HC4LC5) after 24 h and 72 h on poly-HEMA-coated dishes. C33-a CA IX cells incubated in the absence of humanized antibody are marked as “negative control.” **B** Analysis of C33-a CA IX cells by propidium iodide staining and flow cytometry after 72 h of treatment with humanized CA9hu-1 (HC4LC4) and CA9hu-2 (HC4LC5) antibody. C33-a CA IX cells incubated in the absence of humanized antibody are marked as “ctrl”

First, we evaluated the effects of humanized antibodies on the multicellular aggregation of cancer cells during extracellular matrix (ECM) detachment, which represents an efficient mechanism for anoikis inhibition. As it is known detachment of the cells from the extracellular matrix initiates programmed cell death by a process termed anoikis. Malignant cells must acquire anoikis resistance to leave the primary tumor and metastasize. Resistance to anoikis plays a major role in tumor metastasis as tumor cells that survive after detachment from their primary location can travel through circulatory systems. Emerging evidence suggests that as tumor cells lose the requirement for anchorage dependency for growth and survival, they increasingly rely on their ability to adhere to each other (that is, multicellular aggregation) for survival. Figure 6A shows that the humanized antibodies CA9hu-1 (HC4LC4) and CA9hu-2 (HC4LC5) reduce the ability of C33-a CA IX cells to form multicellular aggregates during detached conditions on poly-HEMA coated dishes. To validate the enhanced sensitivity of C33-a CA IX cancer cells treated with the humanized antibody, we performed flow cytometry and propidium iodide staining. Figure 6B shows that antibodies affect the viability of treated cells after 72 h growth in detached conditions. The percentage of dead cells treated with CA9hu-1 (HC4LC4) and CA9hu-2 (HC4LC5) was 37.3% and 35.1%, respectively. In case of C33-a CA IX cells without antibody treatment (“negative control”), we observed only 15.7% ± of dead cells. The data demonstrates the ability of humanized antibody variants to reduce multicellular aggregation of C33-a CA IX-expressing cancer cells and subsequently to enhance their sensitivity to anoikis.

**Fig. 7:**
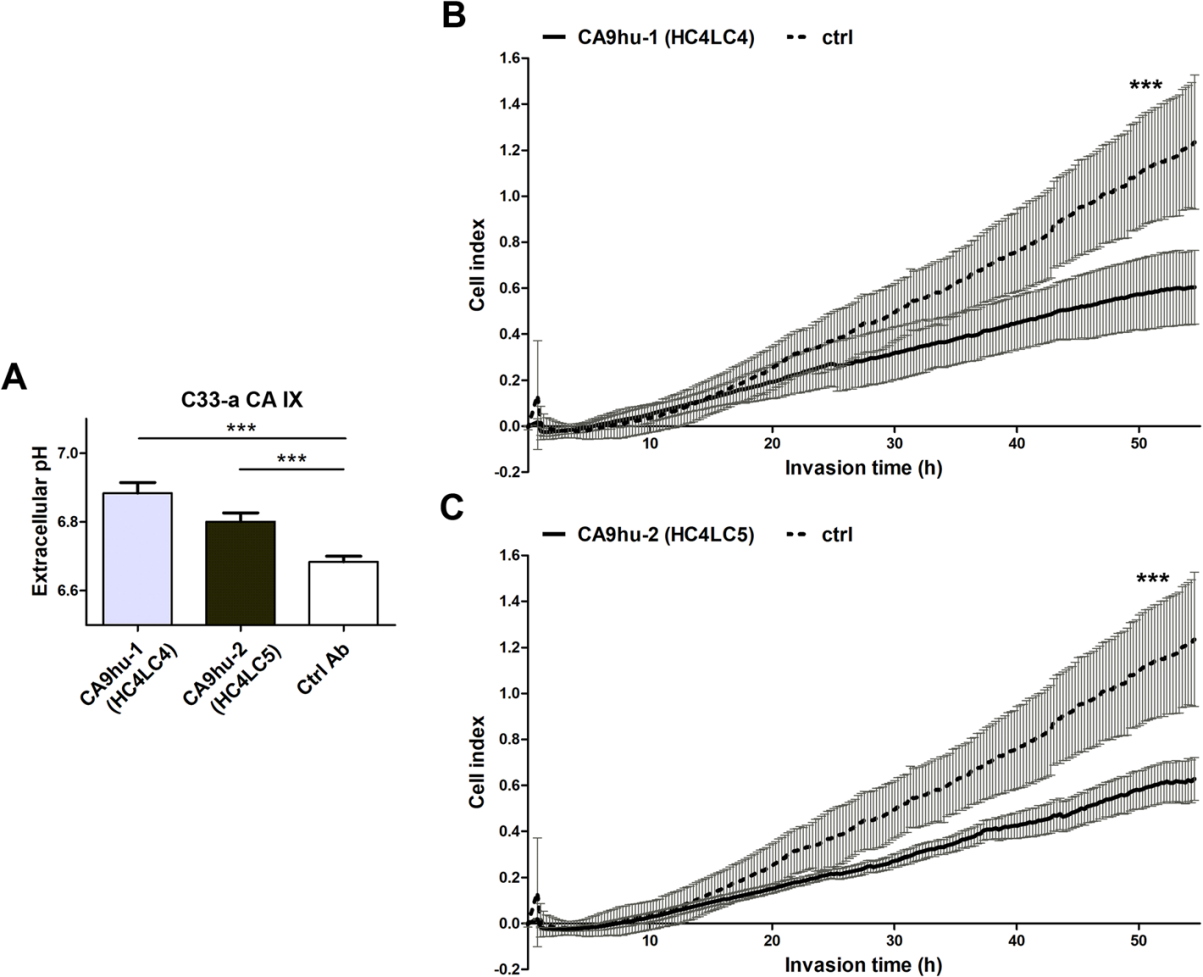
Graphical representation of extracellular pH analyzed in C33-a CA IX (**A**) cells after treatment with CA9hu-1 (HC4LC4) or CA9hu-2 (HC4LC5) for 48 h in hypoxia, *n* = 6, pH values were normalized to cell numbers. **B**, **C** The ability of humanized antibodies to inhibit invasion of cancer cells in comparison with no Ab treatment. The invasion ability of C33-a cells expressing CA IX was significantly reduced after the treatment with both humanized antibody variants. Data in the graph represent the mean ± standard deviation values. Statistical significance of differences was assessed using Student’s *t*-test (*P < 0.05, **P < 0.01, ***P < 0.001), *n* = 6

To validate the ability of humanized antibody variants to affect extracellular acidosis by targeting and blocking CA IX, pH of cell culture media was measured by a non-invasive online pH monitoring using pH measuring device SDR SensorDish Reader. C33-a CA IX cells were grown in a monolayer and incubated in hypoxia (1% O_2_) in the presence of humanized antibodies CA9hu-1 (HC4LC4) or CA9hu-2 (HC4LC5) or control irrelevant IgG1 (50 μg/ml) for 48 h. The results show that treatment with humanized antibody variants led to reduced extracellular acidosis in hypoxic cells Fig. 7A.

To investigate the effect of humanized antibodies on cancer cell invasion, we performed the in vitro xCELLigence cell index impedance measurements using CIM-Plate16 and RTCA DP station. C33-a CA IX cells were resuspended in a serum-free medium in the presence or absence of humanized antibody variant CA9hu-1 (HC4LC4) and CA9hu-2 (HC4LC5). After addition to the matrigel-coated top chamber of the CIM-Plate, C33-a CA IX cells were allowed to migrate towards the bottom chamber containing medium with 10% FCS as a chemoattractant. The foregoing results in Fig. 7B, C demonstrate the ability of the humanized antibody variants to inhibit invasion of CA IX-expressing C33-a cells. Considering the fact that inhibition of cancer cell invasion could lead to limited tumor progression, and consequently, to reduced mortality of cancer patients, this mechanism of action represents an extraordinary beneficial property. Additionally, this is the first demonstration of an aforementioned effect described using PG domain-specific humanized antibodies.

## Discussion

Biological treatment with human or humanized antibodies is one of the most effective and clinically significant therapies that are currently available on the market. Every year, regulators such as EMA or FDA approve increasing numbers of drugs with an antibody basis, and there are already many of them proven and used in clinical practice. In the past decade, there have been approximately fifty new antibodies approved, with a total of more than sixty monoclonal antibodies (mAbs) used in the clinic [33]. Yet, most of the mAbs, either tested in clinical trials or already authorized for the treatment, target the same antigens, and the number of newly developed antibodies directing at novel targets is not so high [34].

Considering the high number of oncogenes and tumor-associated proteins, we can expect the development of many new therapeutics for oncological diseases. This expectation emerges from the significantly lower risk of side effects posed by therapies based on humanized antibodies in comparison with conventional therapeutics, although adverse events can occur [35]. Negative outcomes of clinical development are often related to the ineffectiveness of the immunotherapy rather than a threat to patient’s health, and the failure is often associated with poor patient stratification.

Almost 600 mAb have been examined in clinical trials around the globe since the 1990s, out of which about 30 mAb are now available for cancer therapy. This remarkable progress in cancer treatment has greatly affected the percentage of treatable diseases in practically all types of tumors. The cancer research is now facing another challenge—how to approach the remaining cases where available therapy is still failing? How to treat, for example, TNBC patients that become resistant to HER2-directed treatment?

The most aggressive tumors are often hypoxic. Targeted therapy against these hypoxic tumors must therefore be the highest priority in the near future. The common feature of hypoxic tumors is their resistance to chemotherapy and radiotherapy. Furthermore, hypoxic tumors often do not respond to immunotherapy due to several factors such as acidic TME, increased invasiveness, metastases, inadequate immune response, genomic instability, metabolic reprogramming, or vascularization. The gap in the treatment of hypoxic tumors may lie in the lack of antibodies targeting HIF-1-dependent genes that are upregulated in response to hypoxia and mediate pro-survival pathways.

Carbonic anhydrase IX belongs to a broad group of HIF-1 targets. It is currently one of the best markers of poor prognosis related to hypoxia. Low O_2_ levels do not only regulate CA IX expression on the transcription level, but also affect its enzymatic activity and splicing (important for the correct localization and catalytic activity). Another compelling advantage of CA IX is its easy accessibility given the localization on the plasma membrane. CA IX is key to pH regulation in tumors that increases the acidity of the tumor microenvironment and plays an important role in the invasiveness of tumor cells. Furthermore, CA IX expression is strongly associated with tumor phenotype. All these attributes make CA IX an attractive therapeutic target.

To date, several mAbs against human CA IX have been extensively studied. Most of the studies including the clinical trials were performed in clear cell renal cell carcinomas (ccRCC) models and patients using the chimeric monoclonal antibody G250 known under commercial names RENCAREX® or GIRENTUXIMAB. In most ccRCC tumors, CA IX is frequently expressed at high levels due to the functional inactivation of the VHL tumor suppressor gene that generates defective pVHL protein unable to negatively regulate HIF-1α [36]. Monoclonal antibody G250 and its humanized, chimeric, and bispecific variants were systematically studied in pre-clinical ccRCC models and in clinical cohorts of ccRCC patients [37–40]. This antibody showed good safety, tolerability, and promising efficacy profile in phase I and II clinical trials with more than 100 patients with metastatic RCC [41, 42]. Phase III clinical trial (ARISER), targeted at patients with non-metastatic renal cell carcinoma, showed no significant improvement of disease-free survival among randomized/non-stratified patients treated with RENCAREX® compared to placebo. However, more careful, but retrospective analysis of the data showed that patients with a high tumor CA IX scores have prolonged disease-free survival of about 22 months [43].

In addition, several human CA IX-specific monoclonal antibodies directed to the catalytic domain have been described, but their characterization did not go beyond the preclinical phase [44, 45]. On the other hand, catalytic domain-specific human BAY79-4620 antibody showed potent antitumor efficacy in xenograft models but failed in an early clinical trial due to inadequate toxicity caused by toxin conjugated via self-cleavable linker [46]. Thus, except GIRENTUXIMAB, which would require re-evaluation in improved trial settings, there is currently no CA IX-directed therapeutic antibody under clinical development.

Thus, CA9hu-1 and CA9hu-2 humanized antibodies appear to be promising candidates to fulfill this unmet need. The CA9hu-1 humanized antibody recognizes the exofacial catalytic domain of the CA IX protein. It has several unique characteristics that predetermine its strong anti-cancer effect: high affinity and specificity, absence of cross-reaction with other carbonic anhydrases (a feature that small molecule inhibitors lack), capacity to internalize, and ability to block acidification and retard the tumor growth.

The CA9hu-2 antibody is exceptional due to its binding to the linear epitope in the N-terminal proteoglycan domain of CA IX. After binding to the epitope, the parental murine antibody is able to block the attachment of tumor cells to the extracellular matrix, leading to the decrease of metastases. Similar to CA9hu-1, CA9hu-2 antibody decreases extracellular acidification and does not cross-react with other carbonic anhydrases that play important roles in non-pathological conditions.

Most importantly, both antibodies exhibit the ability to induce ADCC and ADCP activities, which allows us to use them in clinical practice as effective single agents without the need for antibody-drug conjugate.

The humanized antibodies are leading drugs in immunotherapy mainly due to their optimal structure preventing undesired HAMA effects. Therefore, it is predictable that humanized antibodies against CA IX will be in some aspects more efficient than chimeric [47].

## Conclusions

In summary, this investigation has demonstrated that CA9hu-1 and CA9hu-2 humanized antibodies are highly specific to hypoxia-induced CA IX cancer biomarker. They are able to interfere with the function of CA IX in tumor biology and are also capable of engaging innate immune effector mechanisms involved in killing tumor cells. These findings provide the supporting rationale for the further preclinical investigation and subsequent clinical development of these humanized antibodies as immunotherapeutic drugs for patients with solid tumors expressing CA IX.

## Supplementary Information

**Additional file 1 Fig. S1.** Reactivity of twenty-five CA9hu-1 (**A**) and CA9hu-2 (**B**) variants with either CA IX-positive (C33-a CA IX) or CA IX-negative (C33-a neo) antigen determined via ELISA. Samples containing only antibody diluent are marked as “no Ab”. Parental VII/20 (A) / IV/18 (B) as well as chimeric HC0LC0 antibodies were used as reference samples. Data in the graph represent mean ± standard deviation values, *n* = 2. **C** ELISA reactivity of CA9hu-1 and CA9hu-2 variants with FL CA IX and its deletion variants ΔPG and ΔCA expressed in transfected C33-a cells, *n* = 2.

**Additional file 2 Table 1.** SPR data from the analysis of twenty-five CA9hu-1 and CA9hu-2 variants expressed as K_D_ values. Chimeric HC0LC0 antibodies HC0LC0 antibodies (having the murine variable domains and the human Ig constant domains) were used as reference samples.

## Data Availability

The datasets used and/or analyzed during the current study are available from the corresponding author on reasonable request.

## Abbreviations

ADCC: Antibody-dependent cellular cytotoxicity
ADPC: Antibody-dependent cell-mediated phagocytosis
CA9hu-1;2: Carbonic anhydrase 9 humanized-1, 2 antibody
CA: Carbonic anhydrase
CA IX: Carbonic anhydrase IX
ccRCC: Clear cell renal cell carcinomas
CDC: Complement dependent cytotoxicity
CDR: Complementarity-determining region
EC50: Half maximal effective concentration
HAMA: Human anti-mouse antibody response
HC: Antibody heavy chain
HIF(-1): Hypoxia-inducible factor (1)
IMGT: ImMunoGeneTics information system
LC: Antibody light chain
mAb: Monoclonal antibodies
NFAT: Nuclear factor of activated T-cells
PG: Proteoglycan-like domain
RLU: Relative luminescence units
SPR: Surface plasmon resonance
TME: Tumor microenvironment
TNBC: Triple-negative breast cancer
VHL: von Hippel-Lindau
V_H_: Heavy variable domain
V_L_: Light variable domain
