# Supplementary figures and images for: Novel humanized monoclonal antibodies for targeting hypoxic human tumors via two distinct extracellular domains of carbonic anhydrase IX
Source: Cancer Metab. 2022 Feb 2;10:3. doi: 10.1186/s40170-022-00279-8 (PMC8811981; doi:10.1186/s40170-022-00279-8)

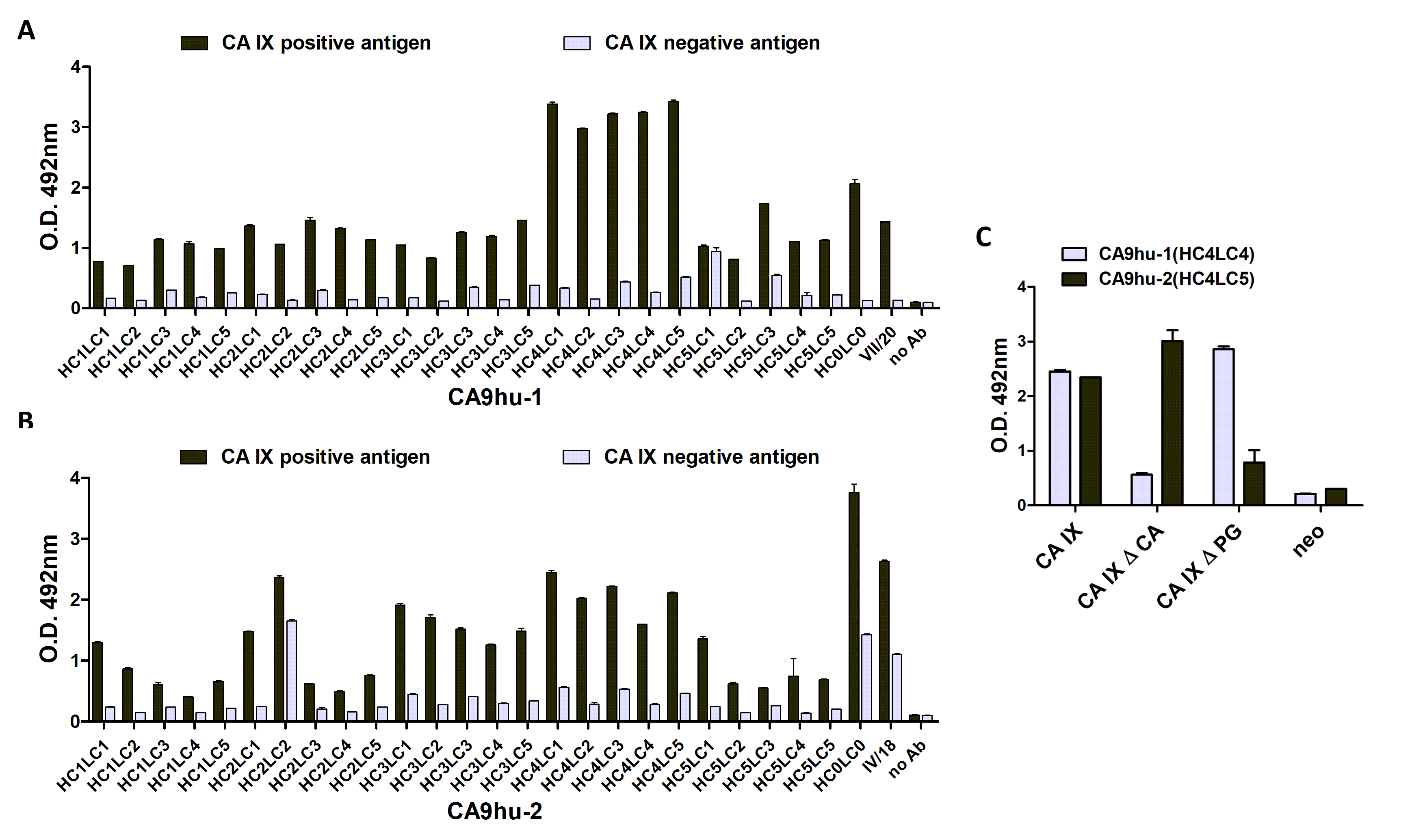

Supplement: Supplementary file 1 — Additional file 1 Fig. S1. Reactivity of twenty-five CA9hu-1 (A) and CA9hu-2 (B) variants with either CA IX-positive (C33-a CA IX) or CA IX-negative (C33-a neo) antigen determined via ELISA. Samples containing only antibody diluent are marked as “no Ab”. Parental VII/20 (A) / IV/18 (B) as well as chimeric HC0LC0 antibodies were used as reference samples. Data in the graph represent mean ± standard deviation values, n = 2. C ELISA reactivity of CA9hu-1 and CA9hu-2 variants with FL CA IX and its deletion variants ΔPG and ΔCA expressed in transfected C33-a cells, n = 2. [file 40170_2022_279_MOESM1_ESM.png]
