# Supplementary material for: Novel humanized monoclonal antibodies for targeting hypoxic human tumors via two distinct extracellular domains of carbonic anhydrase IX
Source: Cancer Metab. 2022 Feb 2;10:3. doi: 10.1186/s40170-022-00279-8 (PMC8811981; doi:10.1186/s40170-022-00279-8)
Supplement: Supplementary file 2 — Additional file 2 Table 1. SPR data from the analysis of twenty-five CA9hu-1 and CA9hu-2 variants expressed as KD values. Chimeric HC0LC0 antibodies HC0LC0 antibodies (having the murine variable domains and the human Ig constant domains) were used as reference samples. [file 40170_2022_279_MOESM2_ESM.docx]

|  | **CA9hu-1** | **CA9hu-2** |
| --- | --- | --- |
|  | **K_D_ (M)** | **K_D_ (M)** |
| **HC1LC1** | **9.70E-08** | **4.40E-08** |
| **HC1LC2** | **8.00E-08** | **5.61E-08** |
| **HC1LC3** | **9.60E-08** | **7.05E-08** |
| **HC1LC4** | **1.00E-07** | **7.49E-08** |
| **HC1LC5** | **9.60E-08** | **7.13E-08** |
| **HC2LC1** | **6.00E-08** | **5.39E-08** |
| **HC2LC2** | **5.30E-08** | **8.77E-08** |
| **HC2LC3** | **5.10E-08** | **9.83E-08** |
| **HC2LC4** | **6.00E-08** | **6.01E-08** |
| **HC2LC5** | **5.60E-08** | **6.05E-08** |
| **HC3LC1** | **9.10E-08** | **3.74E-08** |
| **HC3LC2** | **8.10E-08** | **4.01E-08** |
| **HC3LC3** | **8.20E-08** | **6.23E-08** |
| **HC3LC4** | **9.70E-08** | **7.31E-08** |
| **HC3LC5** | **8.30E-08** | **5.43E-08** |
| **HC4LC1** | **1.52E-08** | **3.96E-08** |
| **HC4LC2** | **9.10E-09** | **3.27E-08** |
| **HC4LC3** | **1.07E-08** | **4.42E-08** |
| **HC4LC4** | **1.52E-08** | **7.46E-08** |
| **HC4LC5** | **1.23E-08** | **4.19E-08** |
| **HC5LC1** | **not detected** | **6.98E-08** |
| **HC5LC2** | **5.20E-08** | **1.05E-07** |
| **HC5LC3** | **5.60E-08** | **5.72E-08** |
| **HC5LC4** | **5.40E-08** | **4.73E-08** |
| **HC5LC5** | **6.40E-08** | **6.51E-08** |
| **HC0LC0** | **2.20E-08** | **1.31E-08** |
